# The contribution of low Apgar scores in identifying neonates with short-term morbidities in a large single center cohort

**DOI:** 10.1038/s41372-024-01944-0

**Published:** 2024-03-28

**Authors:** Samuel Huang, Miheret Yitayew, Henry J. Rozycki

**Affiliations:** 1 School of Medicine, Virginia Commonwealth University, Children’s Hospital of Richmond at VCU, Richmond, VA USA; 2 Division of Neonatal Medicine, Children’s Hospital of Richmond at VCU, Richmond, VA USA

**Keywords:** Risk factors, Translational research

## Abstract

**Objective:**

To evaluate the association and utility of low 1- and 5-min Apgar scores to identify short-term morbidities in a large newborn cohort.

**Methods:**

15,542 infants >22 weeks gestation from a single center were included. Clinical data and low Apgar scores were analyzed for significance to ten short-term outcomes and were used to construct Receiver Operating Characteristic Curves and the AUC calculated for ten outcomes.

**Results:**

A low Apgar score related to all (1-min) or most (5-min) outcomes by univariate and multivariate logistic regression analysis. Including any of the 4 low Apgar scores only improved the clinical factor AUC by 0.9% ± 2.7% (±SD) and was significant in just 5 of the 40 score/outcome scenarios.

**Conclusion:**

The contribution of a low Apgar score for identifying risk of short-term morbidity does not appear to be clinically significant.

## Introduction

The Apgar scoring system was developed by Dr. Virginia Apgar over 70 years ago as a tool to assess the condition of a newborn at birth based on five variables: heart rate, respiratory effort, muscle tone, reflex irritability, and color. Its original purpose was to allow for immediate observation and prompt identification of newborns who need resuscitative measures during transition to extrauterine life [1]. However, over the ensuing seven decades, despite the advancement made in evidence-based newborn resuscitation and the advent of Neonatal Resuscitation Program (NRP) that requires evaluation of the newborn infant without any role for the Apgar score [2], its international recognition and universal use continues.

Over those years, the use of the Apgar scoring tool has gone far beyond its original purpose to guide clinical management decisions and establish a correlation to long term infant health outcomes [3–6]. In a review of 501 papers published in 2018–19, the Apgar score was used as a prognostic factor for outcomes in 19%, more than half of these focused on short term morbidities [7]. Numerous studies have examined the association between low Apgar score and a variety of short-term neonatal morbidities [8–10] but the significance and value of a low Apgar score in identifying newborn infants likely to manifest these morbidities has not been systematically examined.

## Methods

We conducted a retrospective study using data from the medical records of infants born at >22 weeks gestational age at VCU Health Systems in Richmond VA, a regional academic medical center with a 40 bed NICU and an average of 2600 live births per year in the period between 9/1/2013 and 3/30/2020. The Labor and Delivery service maintained a database of live births, including medical record number, mode of delivery, and Apgar scores. The electronic medical record (EMR) for the mothers and newborns of each delivery were then queried for demographic information and all discharge diagnoses. Information contained in both the database and the EMR (birthdate, medical record numbers) was used to confirm matching of each data set.

Ten short term outcomes, defined as occurring during the initial hospital stay, were selected. Each was counted using any of the ICD9 (before 2016) and ICD10 codes that might be applied. For example, in ICD10, Respiratory Distress of the Newborn is coded by either P22.0 or P22.9. The selected common morbidities were as follows: bronchopulmonary dysplasia (BPD); necrotizing enterocolitis (NEC); intraventricular hemorrhage (IVH); retinopathy of prematurity (ROP); hypoxic ischemic encephalopathy (HIE); respiratory distress syndrome (RDS); transient tachypnea of the newborn (TTNB); newborn sepsis, hypoglycemia (HypoG); and meconium aspiration syndrome (MAS).

Eight predictor variables that would be available during the initial hospitalization and that have been previously associated with one or more of the short-term outcomes were recorded for each mother-infant dyad. These included gestational age, birthweight, gender, race, mode of delivery, being small for gestational age (SGA) and 1- and 5-min Apgar scores.

The study was approved as exempt by the Virginia Commonwealth University IRB.

### Analysis

Because of the well-documented variability in Apgar scoring across countries [11], individuals [12] and by newborn conditions [13], we chose to use cut-off scores at both one and 5 min. The most common definition of significance in the 2018–19 review was a total score of less than or equal to 6 [7] while a score of less than or equal to 3 has been commonly used to identify possible asphyxia [14]. We therefore defined four Apgar score groups: 1 L (1 min ≤ 6), 1VL (1 min ≤ 3), 5 L (5 min ≤ 6), and 5VL (5 min ≤ 3) We did not use any scores beyond 5 min because it was generally only done when the previous scores were low, which creates a likely selection bias for this data.

The sensitivity, specificity, negative (NPV) and positive (PPV) predictive value as well as odds ratio for the four low Apgar scores were calculated for each short-term morbidity. Multivariable logistic analysis was performed for each morbidity using each of the low Apgar scores and gestational age by week, gender, race, mode of delivery, and whether they were SGA. Odds ratios, 95% confidence intervals, and *p* values were calculated for all models. Receiver operator characteristic (ROC) curves for the multivariable models were calculated with and without each low Apgar score and the differences in the area under the curve (AUC) when each was included or omitted was calculated. For comparison, a similar analysis was done using the Apgar score first and then adding in the clinical factors. Statistical significance between these AUCs was calculated DeLong’s test [15].

## Results

**Fig. 1:**
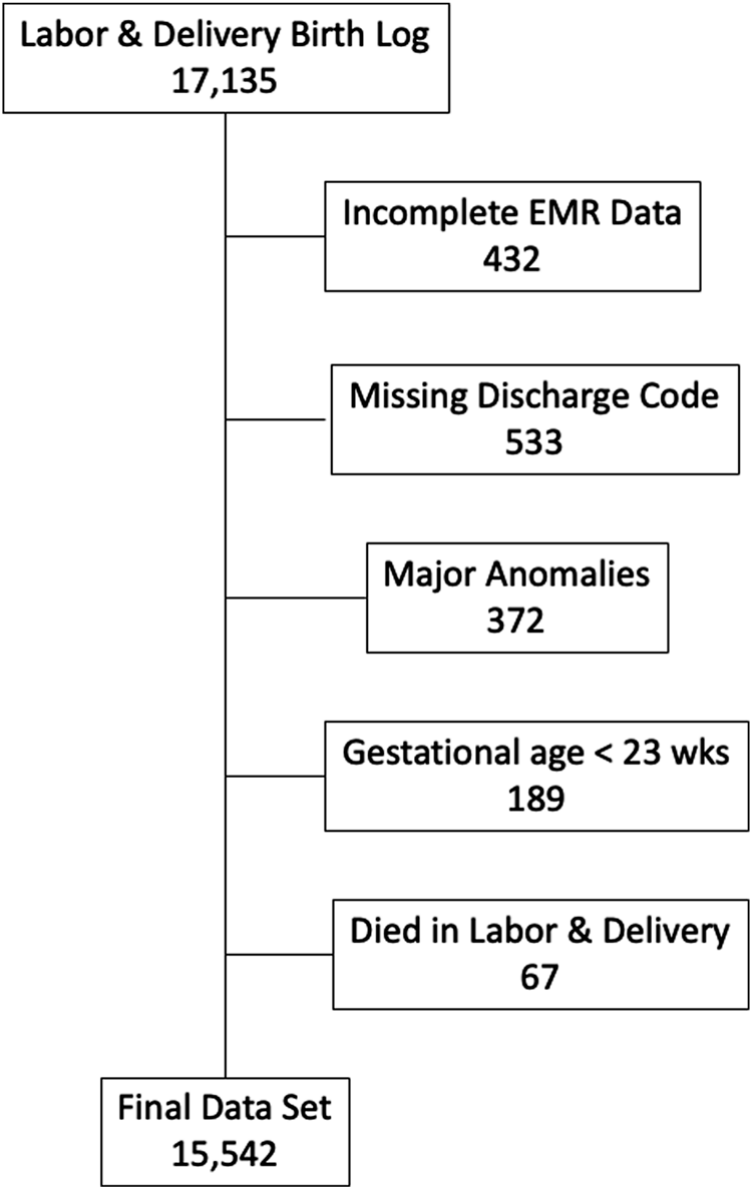
Consort diagram for study. Of the 17,135 deliveries listed in the service database, 905 were eliminated due primarily to missing data leaving the 15,542 subject data set.

Figure 1 is a consort diagram of the study with 17,135 mothers in the labor and delivery birth delivery database from September 1, 2013 to March 30, 2020. Of these 16,703 had complete EMR data for the newborns. There were 533 with missing discharge diagnosis codes, 372 with a variety of congenital anomalies that might impact the outcomes, 189 who were <23 weeks gestation, and 67 who died in the delivery room. The final cohort consisted of 15,542 (90.7%) infants. The median length of stay was 2 days (IQR 2–3 days).

Table 1 presents the incidence of each short-term outcome for the study cohort, ranging from 7.3% for MAS to 0.4% for NEC overall and the incidence of each potential risk factor for each outcome. By univariate analysis of clinical factors, gender was a significant risk for TTNB, RDS, and HypoG, race for all outcomes except HIE, gestational age for all outcomes, mode of delivery for all outcomes except MAS, and SGA status significantly related to TTNB, HypoG, and IVH. Focusing on the Apgar score, as noted in Table 2, the four different low Apgar scores were significantly associated with the ten outcomes in 38 of the 40 scenarios. The only exceptions were TTNB and HypoG for 5VL.

**Table 1:** Demographics and Low Apgar Score distribution for all subjects and short-term outcome (%).

	Total	Sepsis	TTNB	RDS	MAS	BPD	HIE	HypoG	NEC	IVH	ROP
*n*	15542	172 (1.11%)	1065 (6.85%)	734 (4.72%)	1131 (7.28%)	96 (0.62%)	73 (0.47%)	608 (3.91%)	57 (0.37%)	128 (0.82%)	207 (1.33%)
Gender											
Female	7504 (48.3)	77 (44.8)	417 (39.2)	315 (42.9)	549 (48.5)	47 (49.0)	27 (37.0)	260 (42.8)	23 (40.4)	59 (46.1)	97 (46.9)
Male	8038 (51.7)	95 (55.2)	648 (60.8)	419 (57.1)	582 (51.5)	49 (51.0)	46 (63.0)	348 (57.2)	34 (59.6)	69 (53.9)	110 (53.1)
Race											
White	5110 (32.9)	33 (19.2)	341 (32.0)	249 (33.9)	340 (30.1)	22 (22.9)	24 (32.9)	177 (29.1)	10 (17.5)	30 (23.4)	52 (25.1)
African American	5222 (33.6)	106 (61.6)	426 (40.0)	337 (45.9)	404 (35.7)	56 (58.3)	34 (46.6)	270 (44.4)	41 (71.9)	79 (61.7)	122 (58.9)
Other	5210 (33.5)	33 (19.2)	298 (28.0)	148 (20.2)	387 (34.2)	18 (18.8)	15 (20.5)	161 (26.5)	6 (10.5)	19 (14.8)	33 (15.9)
Birth Weight (kg)	3.212 (±0.625)	2.988 (±0.976)	3.156 (±0.703)	3.01 (±0.909)	3.185 (±0.666)	2.722 (±1.209)	2.798 (±0.993)	3.153 (±0.677)	2.66 (±1.185)	2.965 (±1.034)	2.901 (+0.1)
SGA	1025 (6.6)	8 (4.6)	46 (4.3)	82 (11.2)	1 (<1)	6 (6.3)	3 (4.1)	77 (12.7)	3 (5.2)	12 (9.4)	12 (5.8)
Gestational Age											
>37 weeks	13,719 (88.3)	66 (38.4)	820 (77.0)	216 (29.4)	1060 (93.7)	3 (3.1)	56 (76.7)	353 (58.1)	3 (5.3)	21 (16.4)	1 (0.5)
33–36 weeks	1285 (8.3)	18 (10.5)	195 (18.3)	178 (24.3)	60 (5.3)	5 (5.2)	15 (20.5)	179 (29.4)	9 (15.8)	13 (10.2)	4 (1.9)
<33 weeks	538 (3.5)	88 (51.2)	50 (4.7)	340 (46.3)	11 (1.0)	88 (91.7)	2 (2.7)	76 (12.5)	45 (78.9)	94 (73.4)	202 (97.6)
Delivery											
Vaginal Delivery	11,496 (74.0)	80 (46.5)	646 (60.7)	319 (43.5)	815 (72.1)	38 (39.6)	30 (41.1)	367 (60.4)	17 (29.8)	75 (36.2)	52 (40.6)
C-Section	3917 (25.2)	91 (52.9)	403 (37.8)	390 (53.1)	315 (27.9)	59 (61.5)	43 (58.9)	236 (38.8)	40 (70.2)	121 (58.5)	72 (56.2)
Apgar											
1VL	699 (4.5)	55 (32.0)	91 (8.5)	199 (27.1)	127 (11.2)	41 (42.7)	61 (83.6)	68 (11.2)	23 (40.4)	58 (45.3)	69 (33.3)
1L	1661 (10.7)	97 (56.4)	228 (21.4)	403 (54.9)	267 (23.6)	76 (79.2)	67 (91.8)	138 (22.7)	44 (77.2)	92 (71.9)	140 (67.6)
5VL	121 (0.8)	11 (6.4)	5 (0.5)	37 (5.0)	17 (1.5)	9 (9.4)	19 (26.0)	6 (1.0)	6 (10.5)	20 (15.6)	12 (5.8)
5L	482 (3.1)	60 (34.9)	61 (5.7)	194 (26.4)	68 (6.0)	43 (44.8)	51 (69.9)	50 (8.2)	24 (42.1)	62 (48.4.)	72 (34.8)

**Table 2:** Sensitivity, Specificity, Positive (PPV) and Negative (NPV) Predictive Values and Odds Ratio for Low Apgar Scores for discharge diagnosis outcomes.

Morbidity	Sepsis	TTNB	RDS	MAS	BPD	HIE	HypoG	NEC	ROP	IVH
APGAR 1VL										
Sensitivity	0.325	0.086	0.276	0.112	0.441	0.836	0.112	0.426	0.337	0.46
Specificity	0.958	0.958	0.966	0.96	0.957	0.959	0.958	0.956	0.959	0.958
PPV	0.079	0.13	0.285	0.182	0.059	0.087	0.097	0.033	0.099	0.083
NPV	0.992	0.934	0.965	0.932	0.996	0.999	0.964	0.998	0.991	0.995
Odds Ratio (Confidence Interval)	11.01* (7.85–15.26)	2.14* (1.69–2.67)	10.87* (9.01–13.08)	3.06* (2.49–3.73)	17.68* (11.60–26.78)	117.90* (65.55–230.93)	2.86* (2.18–3.71)	16.22* (9.31–27/86)	11.82* (8.71–15.90)	19.62* (13.67–28.07)
APGAR 1L										
Sensitivity	0.574	0.215	0.558	0.236	0.817	0.918	0.228	0.815	0.683	0.73
Specificity	0.898	0.901	0.915	0.903	0.897	0.897	0.898	0.895	0.901	0.898
PPV	0.058	0.137	0.243	0.161	0.046	0.04	0.083	0.026	0.084	0.055
NPV	0.995	0.94	0.977	0.938	0.999	1	0.966	0.999	0.995	0.998
Odds Ratio (Confidence Interval)	11.87* (8.72–16.21)	2.49* (2.12–2.90)	13.59* (11.61–15.92)	2.89* (2.49–3.34)	39.01* (23.60–68.36)	96.96* (45.65–250.8)	2.60* (2.13–3.15)	37.65* (19.73–79.39)	19.52* (14.54–26.47)	23.82* (16.19–35.89)
APGAR 5VL										
Sensitivity	0.067	0.005	0.052	0.015	0.099	0.279	0.01	0.115	0.059	0.159
Specificity	0.993	0.992	0.994	0.993	0.993	0.993	0.992	0.993	0.993	0.993
PPV	0.091	0.041	0.306	0.14	0.074	0.157	0.05	0.05	0.099	0.165
NPV	0.99	0.932	0.956	0.928	0.995	0.997	0.961	0.997	0.988	0.993
Odds Ratio (Confidence Interval)	9.84* (4.91–17.86)	0.59 (0.21–1.30)	9.55* (6.37–14.05)	2.10* (1.212–3.43)	14.92* (6.84–28.92)	58.01* (32.32–100.52)	1.30 (0.51–2.72)	17.31* (6.52–38.35)	8.76* (4.51–15.54)	28.40 *(16.54–46.70)
APGAR 5L										
Sensitivity	0.364	0.058	0.272	0.061	0.473	0.75	0.084	0.462	0.356	0.492
Specificity	0.972	0.971	0.98	0.971	0.971	0.972	0.971	0.97	0.973	0.973
PPV	0.124	0.127	0.402	0.141	0.089	0.106	0.104	0.05	0.149	0.129
NPV	0.993	0.934	0.965	0.93	0.997	0.999	0.963	0.998	0.991	0.996
Odds Ratio (Confidence Interval)	20.10* (14.36–27.90)	2.04* (1.53–2.67)	18.76* (15.32–22.95)	2.17* (1.65–2.80)	30.40* (19.87−46.38)	103.92* (60.79–186.62)	3.05* (2.27–4.10)	27.92* (15.95–48.52)	20.02* (14.71–27.05)	34.33* (23.87–49.26)

*p > 0.05.

To examine whether a low Apgar score remained a significant risk factor when other clinical risk factors (gestational age, gender, race, mode of delivery, and SGA status) were accounted for, multivariable logistic models were created with each of the four low Apgar scores for each outcome. As shown in Table 3, in this analysis, there were 11 scenarios where a low score was not significant. For HypoG, only 5VL remained significant, and for NEC, none of the Apgar scores remained significant. In addition, a 1L score was not significant for BPD or ROP, a 5VL score was not significant for IVH and the 5L score was not significant for TTNB.

**Table 3:** Odds ratio for Low Apgar Score in multivariable logistic models for each short-term outcome. Other variables were gestational age, gender, race, mode of delivery, and SGA status.

	Outcome	Sepsis	TTNB	RDS	MAS	BPD	HIE	HypoG	NEC	ROP	IVH
Apgar 1VL	Odds Ratio	1.725	1.368	2.773	4.25	0.511	113.611	1.072	0.741	0.279	1.945
	95% Confidence Interval	(1.095–2.681)	(1.057–1.752)	(2.088–3.657)	(3.401–5.282)	(0.28–0.921)	(60.838–228.894)	(0.771–1.468)	(0.364–1.495)	(0.165–0.466)	(1.177–3.189)
	*p* value	0.015	0.015	*p* < 0.001	*p* < 0.001	0.027	*p* < 0.001	0.687	0.405	*p* < 0.001	0.009
Apgar 1L	Odds Ratio	2.903	1.868	4.411	3.804	1.075	85.288	1.173	2.534	0.628	3.279
	95% Confidence Interval	(1.885–4.424)	(1.565–2.221)	(3.588–5.411)	(3.242–4.454)	(0.49–2.405)	(39.294–223.505)	(0.908–1.501)	(0.963–6.806)	(0.373–1.044)	(1.862–5.748)
	*p* value	*p* < 0.001	*p* < 0.001	*p* < 0.001	*p* < 0.001	0.86	*p* < 0.001	0.219	0.061	0.075	*p* < 0.001
Apgar 5VL	Odds Ratio	0.414	0.25	0.143	5.219	0.148	60.807	0.176	0.459	0.041	0.842
	95% Confidence Interval	(0.187–0.85)	(0.086–0.571)	(0.077–0.266)	(2.851–9.14)	(0.059–0.34)	(29.783–120.605)	(0.066–0.394)	(0.156–1.171)	(0.018–0.089)	(0.425–1.623)
	*p* value	0.021	0.003	*p* < 0.001	*p* < 0.001	*p* < 0.001	*p* < 0.001	*p* < 0.001	0.124	*p* < 0.001	0.613
Apgar 5L	Odds Ratio	2.324	1.129	2.715	4.031	0.482	123.458	0.688	0.825	0.224	2.036
	95% Confidence Interval	(1.436–3.73)	(0.816–1.537)	(1.97–3.715)	(3–5.355)	(0.263–0.876)	(68.599–231.281)	(0.461–1.009)	(0.396–1.715)	(0.131–0.378)	(1.207–3.424)
	*p* value	0.001	0.456	*p* < 0.001	*p* < 0.001	0.017	*p* < 0.001	0.06	0.605	*p* < 0.001	0.008

**Fig. 2:**
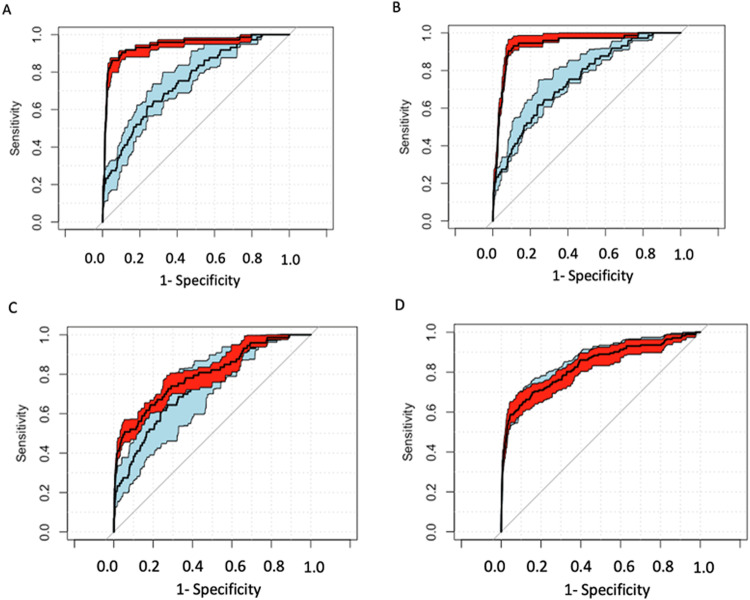
ROC curves for multivariable logistic regression models. **A** HIE without (blue/light gray) and with (red/dark gray) 1VL. **B** HIE without (blue/light gray) and with (red/dark gray) 1L. **C** Sepsis without (blue/light gray) and with (red/dark gray) 1VL. **D** Sepsis without (blue/light gray) and with (red/dark gray)1L.

To further define how much a low Apgar score contributes to the risk identification of newborn infants for the ten outcome diagnoses, ROC curves were created for each diagnosis using multivariate equations incorporating the clinical factors listed above. with and without each of the 4 low Apgar scores, and the significance of the difference in the AUC when the low Apgar was present or absent determined. Birthweight was not included as birthweight and SGA status were, in combination, stronger contributors to the final model. We also examined the AUC for the ROC curves created using the Apgar score first and then adding the clinical factors. In Tables 4 and 5, for each Apgar score, the upper rows are for the condition where the score is added to the ROC constructed from the clinical factors, while the second group of rows shows what happens to the AUC when the clinical factors are added to the model constructed first with the Apgar score. When the 1 min Apgar score was added (Table 4), the AUC increased significantly for HIE at both the L and VL levels and for RDS and MAS for a score <6. There was no effect for the other 17 outcomes. The average change in AUC was 3.92% (CI 0.60 to 7.25). In contrast, adding the clinical factors to the Apgar score curve increased the AUC significantly for all the outcomes and far more substantially (average 27.64% CI 22.81 to 32.47). The results for the 5 min L and VL scores (Table 5) are similar. Adding 5-minute scores to clinical factors changed the AUC of the ROCs by 1.836% (CI −0.3593 to 4.031) while adding clinical factors to the 5 min score increased the AUCs by 45.01% (CI 36.93 to 53.09). Figure 2 illustrates the difference in the ROC curves when the addition of a low Apgar is significant (HIE) and when it is not (sepsis).

**Table 4:** AUC of ROC of clinical factors without and with low Apgar scores (top 4 rows of 1VL and 1L sections) and for ROC of low Apgar score without and with clinical factors (bottom 4 rows of 1VL and 1L sections).

	Outcome	Sepsis	TTNB	RDS	MAS	BPD	HIE	HypoG	NEC	ROP	IVH
APGAR 1VL	AUC Clinical Factors	0.836	0.644	0.883	0.647	0.980	0.749	0.744	0.972	0.992	0.944
	AUC Clinical Factors + Apgar	0.845	0.649	0.901	0.669	0.979	0.944	0.745	0.972	0.991	0.958
	% difference	1.024	0.801	2.030	3.432	0.067	26.091	0.092	0.043	0.037	1.460
	*P* value	0.744	0.671	0.091	0.059	0.959	0.000	0.962	0.974	0.769	0.316
	AUC Apgar	0.646	0.522	0.626	0.537	0.706	0.536	0.705	0.649	0.712	0.646
	AUC Apgar + Clinical Factors	0.845	0.649	0.901	0.669	0.979	0.745	0.972	0.991	0.958	0.845
	% difference	30.776	24.294	44.001	24.547	38.652	39.013	37.850	52.624	34.523	30.776
	*P* value	0.000	0.000	0.000	0.000	0.000	0.000	0.000	0.000	0.000	0.000
APGAR 1L	AUC Clinical Factors	0.836	0.644	0.883	0.647	0.980	0.749	0.744	0.972	0.992	0.944
	AUC Clinical Factors + Apgar	0.865	0.662	0.916	0.683	0.980	0.937	0.746	0.976	0.992	0.963
	% difference	3.437	2.804	3.682	5.575	0.004	25.152	0.307	0.390	0.008	2.028
	*P* value	0.249	0.135	0.001	0.002	0.997	0.000	0.874	0.753	0.949	0.146
	AUC Apgar	0.738	0.859	0.739	0.570	0.859	0.563	0.859	0.792	0.815	0.738
	AUC Apgar + Clinical Factors	0.865	0.980	0.916	0.683	0.980	0.746	0.976	0.992	0.963	0.865
	% difference	17.185	14.145	23.883	19.689	14.145	32.461	13.643	25.207	18.224	17.185
	*P* value	0.000	0.000	0.000	0.000	0.000	0.000	0.000	0.000	0.000	0.000

**Table 5:** AUC of ROC of clinical factors without and with low Apgar scores (top 4 rows of 5VL and 5L sections) and for ROC of low Apgar score without and with clinical factors (bottom 4 rows of 5VL and 5L sections).

	Outcome	Sepsis	TTNB	RDS	MAS	BPD	HIE	HypoG	NEC	ROP	IVH
APGAR 5VL	AUC Clinical Factors	0.836	0.644	0.883	0.647	0.980	0.749	0.744	0.972	0.992	0.944
	AUC Clinical Factors + Apgar	0.833	0.644	0.880	0.648	0.980	0.798	0.742	0.972	0.992	0.941
	% difference	0.369	0.000	0.315	0.210	0.019	6.572	0.267	0.010	0.032	0.283
	*P* value	0.908	0.979	0.806	0.908	0.988	0.230	0.892	0.994	0.798	0.873
	AUC Apgar	0.542	0.500	0.531	0.505	0.563	0.507	0.586	0.531	0.576	0.542
	AUC Apgar + Clinical Factors	0.833	0.644	0.880	0.648	0.980	0.742	0.972	0.992	0.941	0.833
	% difference	53.742	28.712	65.784	28.283	74.248	46.420	65.855	86.885	63.493	53.742
	*P* value	0.000	0.000	0.000	0.000	0.000	0.000	0.000	0.000	0.000	0.000
APGAR 5L	AUC Clinical Factors	0.836	0.644	0.883	0.647	0.980	0.749	0.744	0.972	0.992	0.944
	AUC Clinical Factors + Apgar	0.850	0.645	0.896	0.656	0.980	0.915	0.743	0.972	0.991	0.958
	% difference	1.625	0.169	1.507	1.508	0.008	22.159	0.158	0.011	0.025	1.469
	*P* value	0.603	0.929	0.213	0.406	0.995	0.000	0.936	0.993	0.842	0.317
	AUC Apgar	0.674	0.515	0.631	0.517	0.729	0.532	0.733	0.666	0.730	0.674
	AUC Apgar + Clinical Factors	0.850	0.645	0.896	0.656	0.980	0.743	0.972	0.991	0.958	0.850
	% difference	26.057	25.130	42.044	26.981	34.420	39.540	32.602	48.879	31.313	26.057
	*P* value	0.000	0.000	0.000	0.000	0.000	0.000	0.000	0.000	0.000	0.000

## Discussion

The primary and predominant purpose of the Apgar score has been to assess the status of an infant in the first few minutes after birth [1, 7]. The rationale for such a scoring system is based on the understanding that having difficulties in the transition to extrauterine life is not good for the newborn, i.e., that such difficulties are associated with worse outcomes so identifying these babies could lead to interventions that could mitigate these outcomes. This is supported by the observation that the adoption of the Apgar score did not become widespread, and then universal, until there was evidence that low scores occurred far more frequently in babies who either died or had neurological deficits in the first year of life [16, 17].

Over the decades, the score has consistently been used as a risk factor in clinical studies [7]. It has been associated not only with an increased incidence of long-term neurological conditions, including cerebral palsy and seizures [18], but also with a wide variety of conditions such as attention deficit disorder/hyperactivity [19], permanent dentition [20], cancer [21], food allergy [22], autism spectrum disorder [23], polycystic kidney disease [24] and amblyopia [25]. The Apgar score is used as often for research into morbidities that manifest in the post-natal period, including all the discharge diagnoses used as short-term outcomes in this current study [9, 26–34]. Short term outcomes have also been used for all studies that have examined modifications or replacements for the Apgar, and any future such efforts are likely to do the same [35–37]. It is noteworthy that the NRP does not use the 1- or 5-min Apgar score.

This study used a range of morbidities occurring during the initial hospital stay to determine, first, if a low Apgar score is more frequent in those babies who were given these diagnoses compared to those without the conditions and confirmed previous associations for the risk factors and the various short-term morbidities. We confirmed that low scores at 1 or 5 minutes were significantly associated with the outcomes examined. Of the 10 outcomes and 4 low Apgar score groups, low scores were significant in 38 circumstances; the exceptions were the 5VL score for HypoG and TTNB. When other clinical factors were included in the analysis (Table 3), low scores lost their significance in an additional 9 outcome/score groups. 1L was significant for six, 5VL for eight, and 5L for six outcomes respectively.

The AUC value of the ROC is often used to assess the clinical value of a predictive model [38] with higher values above 0.5 indicating a better model. We have used this to further analyze how much the presence of a low Apgar score contributes to identifying newborn infants who will go on to have one of the short-term outcomes included in this study. This confirmed that low Apgar scores can make a major and significant contribution in predicting HIE, which is not surprising since low scores are often part of the diagnosis [4] and supports the validity of this analytic method. Overall, the addition of a low Apgar score added little to the inclusive predictive model. It was only statistically significant for the 1L Apgar score for RDS and MAS. Otherwise, it improved the AUC by less than 3.5%, and in many cases by less than 1%. In contrast, the addition of clinical factors to ROCs constructed by Apgar scores alone increased by 14–86%, indicating that the Apgar score does not contribute as much to identifying newborns at risk for short term morbidities as other clinical factors combined.

There are several significant limitations to this study that should be noted. The study used retrospective data from a single center. The ten outcomes had a wide range of incidences, which can have an effect on predictive values, for example, and several are associated primarily with prematurity, specifically RDS, BPD, ROP, IVH, and NEC, but previous studies have included Apgar scores in risk assessments for these conditions [27, 31, 33]. We chose to include all gestational ages in our analysis because all newborns receive an Apgar score. Additionally, even though the incidence of several of the prematurity-related outcomes is very low in the term subjects, the proportion of cases made up of term infants is high. For example, for RDS, the incidence in term infants was 1.5% compared to 63% of those <33 weeks, but because there were 25 times as many term as very preterm subjects, term infants made up 29% of those with RDS. Prematurity was also a cofactor used in the multivariate analyses to help account for its influence. The accuracy of discharge diagnosis codes has been questioned [39]. As one example, we found several instances where codes for both TTNB and RDS were assigned to the same subject. Our goal was to ensure that all potential cases were captured, so we used a wide range of codes. As a result, for some short-term outcomes, such as MAS, there was a high incidence. Additionally, other risk factors such as maternal age or maternal chorioamnionitis were not included. We chose to use the two most common [7] cut-off values at 1 and 5 minutes rather than all the Apgar scores from 1 to 10 to account for some of the known variability in scoring and capture a sufficient number of subjects per outcome to analyze. Other investigators have used the complete Apgar scale, usually.in long term outcome studies involving over one million subjects [9]. Finally, Dr. Apgar designed her system to assess the status of the infant immediately after birth. Starting in 1966 [16], however, it has been used as a risk factor hundreds of times.

Strengths include a larger number of subjects than most studies which have examined the Apgar score in relation to short term outcomes. While we looked at the common ways of assessing a risk factor, such as sensitivity, specificity, positive and negative predictive value, and the odds ratio within the context of a multivariable analysis as well as the AUC of the ROC graphs, adding the AUC analysis with and without the low Apgar is a way to directly assess the question of its utility.

The Apgar score has been performed around the world to an estimated three billion or more newborn infants over the last 70 years. During that time, concerns have been repeatedly raised about it. Yet it remains an important tool in the delivery room for assessing the immediate condition of the newborn. It appears to have good utility for assessing risks of long-term outcomes when applied to large populations, but our findings suggest that it is not a significant contributor to identifying newborn infants who would benefit from a higher level of care because of the risk of short-term outcomes.

## Data Availability

The datasets generated during and analyzed during the current study are not publicly available due to HIPAA and privacy restrictions, but could be made available from the corresponding author on reasonable request.
